# Is L-methionine a trigger factor for Alzheimer’s-like neurodegeneration?: Changes in Aβ oligomers, *tau* phosphorylation, synaptic proteins, *Wnt* signaling and behavioral impairment in wild-type mice

**DOI:** 10.1186/s13024-015-0057-0

**Published:** 2015-11-21

**Authors:** Cheril Tapia-Rojas, Carolina B. Lindsay, Carla Montecinos-Oliva, Macarena S. Arrazola, Rocio M. Retamales, Daniel Bunout, Sandra Hirsch, Nibaldo C. Inestrosa

**Affiliations:** Departamento de Biología Celular y Molecular; Facultad de Ciencias Biológicas, P. Centro de Envejecimiento y Regeneración (CARE), Pontificia Universidad Católica de Chile, Santiago, Chile; Institute of Nutrition and Food Technology (INTA), Universidad de Chile, Santiago, Chile; Centre for Healthy Brain Ageing, School of Psychiatry, Faculty of Medicine, University of New South Wales, Sydney, Australia; Centro UC Síndrome de Down, Pontificia Universidad Católica de Chile, Santiago, Chile; Centro de Excelencia en Biomedicina de Magallanes (CEBIMA), Universidad de Magallanes, Punta Arenas, Chile; CARE Biomedical Center, Pontificia Universidad Católica de Chile, Av. Alameda 340, Santiago, Chile

**Keywords:** L-Methionine, Amyloid, Tau, Memory impairment, Alzheimer’s disease

## Abstract

**Background:**

L-methionine, the principal sulfur-containing amino acid in proteins, plays critical roles in cell physiology as an antioxidant and in the breakdown of fats and heavy metals. Previous studies suggesting the use of L-methionine as a treatment for depression and other diseases indicate that it might also improve memory and propose a role in brain function. However, some evidence indicates that an excess of methionine can be harmful and can increase the risk of developing Type-2 diabetes, heart diseases, certain types of cancer, brain alterations such as schizophrenia, and memory impairment.

**Results:**

Here, we report the effects of an L-methionine-enriched diet in wild-type mice and emphasize changes in brain structure and function. The animals in our studypresented 1) higher levels of phosphorylated *tau* protein, 2) increased levels of amyloid-β (Aβ)-peptides, including the formation of Aβ oligomers, 3) increased levels of inflammatory response,4) increased oxidative stress, 5) decreased level of synaptic proteins, and 6) memory impairment and loss. We also observed dysfunction of the *Wnt* signaling pathway.

**Conclusion:**

Taken together, the results of our study indicate that an L-methionine-enriched diet causes neurotoxic effects *in vivo* and might contribute to the appearance of Alzheimer’s-like neurodegeneration.

**Electronic supplementary material:**

The online version of this article (doi:10.1186/s13024-015-0057-0) contains supplementary material, which is available to authorized users.

## Background

Methionine and cysteine are considered to be the principal sulfur-containing amino acids in proteins, and they play critical roles in cell metabolism. Methionine aids in the breakdown of fats by both preventing their accumulation in the arteries [1], aiding the digestive system and facilitating the elimination of heavy metals from the body, which can be converted into cysteine to prevent toxic damage in the liver. Methionine is also an important antioxidant because its sulfur group can inactivate free radicals [2–5], and it is one of the three amino acids that the body needs to produce creatine, which is an essential compound for energy production and muscle building [6]. In addition, it may be useful in the treatment of depression [7, 8]; some studies have indicated that methionine might improve memory recall and suggested a key role for this aminoacid in brain processes. Methionine deficiencies can trigger several alterations, such as fatty liver, slow growth, weakness, edema and skin lesions [9, 10]. Conversely, severe methionine deficiency might cause dementia [11]. Although methionine is a key amino acid for humans [12, 13], there is evidence that excessive uptake can become harmful [9, 10].

Methionine metabolism begins with its conversion to homocysteine (Hcy) via its intermediate, S-adenosyl-methionine (SAM). This sequence of reactions is called transmethylation, and it ubiquitously occurs in mammalian cells [14]. Then, Hcy is removed by its combination with serine to produce cystathionine, which is cleaved to form α-ketobutyrate and cysteine. Additionally, homocysteine may be methylated back to methionine by methionine synthase (MS) [15]. High levels of homocysteine are associated with diverse illnesses, such as cardiovascular and cerebrovascular disease, renal dysfunction, and dementia [16–18]. Similarly, a diet rich in methionine, which is an amino acid typically found in fish, beans, red meat, eggs, garlic, lentils, onions, yogurt and seeds, can cause deleterious effects on one’s health [19]. Several researchers have linked the consumption of both unprocessed and processed red meat with a higher risk of developing Type 2 diabetes [20], heart disease, and certain types of cancer [21]. Interestingly, it has been reported that the high consumption of methionine could promote brain damage. In the past, the transmethylation theory of schizophrenia was proposed, based on the fact that methyl donors such as methionine can exacerbate psychotic symptoms [22–25]. Additionally, recent clinical studies link high levels of plasma methionine sulfoxide, an intermediate in the methionine cycle, with ageing and Alzheimer’s disease (AD) [26, 27]. Furthermore, long-term exposure to high levels of methionine induces memory deficit in zebrafish [28]; however, a detailed analysis of the effects of a methionine-rich diet on the hippocampus, a structure essential to learning and memory, has not been carried out in wild-type mice. Overall, although methionine is a key aminoacid in humans [26, 27], a large body of evidence supports the notion that excessive uptake can become harmful, further clarifying that the way we eat is fundamental in a healthy lifestyle.

In the present study, we performed a detailed analysis to determine the effects of a high methionine diet in wild-type mice on hippocampal structure and function. We treated wild-type C57/BL6 mice for 12 weeks with 8.2 g/kg of L-methionine, a dosage twice the concentration found in a normal diet [29, 30]. Then, the animals were subjected to different behavioral tests. Next, brain slices were obtained to evaluate different histopathological markers of AD using both immunohistochemical and biochemical analyses. Our results showed that the brains of mice with a methionine-rich diet presented 1) increased levels of phosphorylated *tau* protein, 2) increased levels of amyloid-β (Aβ) peptides and Aβ oligomers, 3) neuroinflammation, 4) increased levels of nitro-tyrosinated protein, a marker of oxidative stress, 5) decreased levels of pre- and post- synaptic proteins, and 6) memory impairment accompanied by the loss of function of the *Wnt* signaling pathway. Taken together, these results suggest that a methionine-enriched diet triggers neurotoxic effects *in vivo* and might contribute to the appearance of Alzheimer’s-like neurodegeneration.

## Results

Several studies have demonstrated that L-methionine is an important and essential amino acid; however, high levels have been associated with deleterious effects [9, 10]. We treated 2-month-old mice with high doses of L-methionine (8.2 g/kg) administered in their drinking water. This dosage was reported to generate an increase of methionine in plasma without reaching toxic levels [29]. The treatment lasted 12 weeks, and we studied its effects in the mouse brain. The health of the animals during the treatment was closely supervised, the body weight was measured weekly (Additional file 1: Figure S1), and biochemical analysis of the blood was performed after treatment was completed (Additional file 2: Table S1). No significant differences in these parameters were observed between the control and L-methionine-treated mice.

### Chronic treatment with L-methionine induces

#### *Tau* phosphorylation

**Fig. 1:**
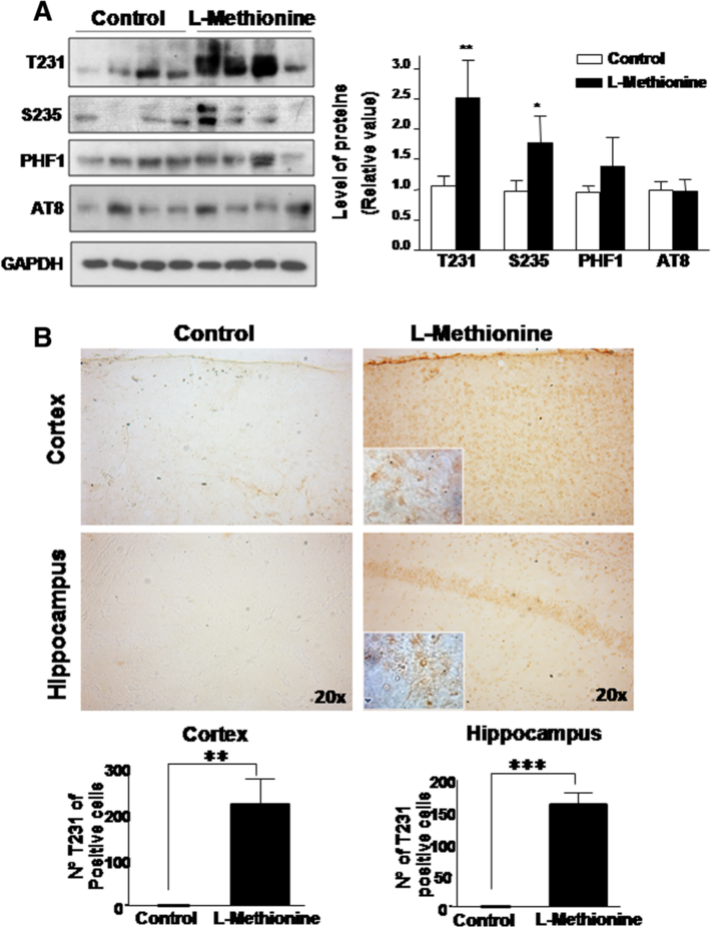
L-methionine treatment increases *tau* phosphorylation in the hippocampus. **a** Different phosphorylation epitopes of *tau * were evaluated in control and L-methionine hippocampal lysates. Each lane represents samples from a different animal. The quantification is shown in the right panel. **b** Immunocytochemistry was used to evaluate the presence of T231-positive cells. (Top left) Cortex and hippocampal sections of control mice; (top right) L-methionine sections at 20x (inset 40x). The diagram below showsthat the respective quantification (*n* ≥ 4) bars are mean ± SEM. *P < 0.05; **P < 0.01, ***P < 0.001

Previous studies have indicated that chronic treatment with methionine inactivates several phosphatases and subsequently induces the phosphorylation of neurofilaments [31], which results in cytoskeleton impairments [32, 33]. Furthermore, it was demonstrated that a high methionine diet increased the levels of *tau* phosphorylation in a mouse model of AD [34]. Therefore, we examined the effect of this type of diet on *tau* protein phosphorylation. In the L-methionine treated group, we observed a significant increase in phosphorylation at two of the four evaluated phosphorylation sites, T231 and S235. No changes were observed in other epitopes with the PHF1 and AT8 antibodies (Fig. 1a). Moreover, we decided to analyze *tau* phosphorylation in distinct brain sections; specifically, both the hippocampus and cortex were examined by immunocytochemistry using the antibody for T231. The results showed that the brains of L-methionine-treated mice had significantly higher levels of T231-positive cells compared with those of control mice in both the hippocampus and cortex (Fig. 1b). Moreover, the same tissues were evaluated with the AT8 antibody, and no significant changes were observed (Additional file 3: Figure S2). Interestingly, the epitopes T231 and S235 in the *tau* protein have been associated with the triggering process of *tau* aggregation, which later constitutes neurofibrillary tangles [35]. Therefore, these results suggest that high levels of methionine favor *tau* phosphorylation and may induce the dissociation of this protein from microtubules to begin its auto-aggregation process.

### Accumulation of Aβ peptides

**Fig. 2:**
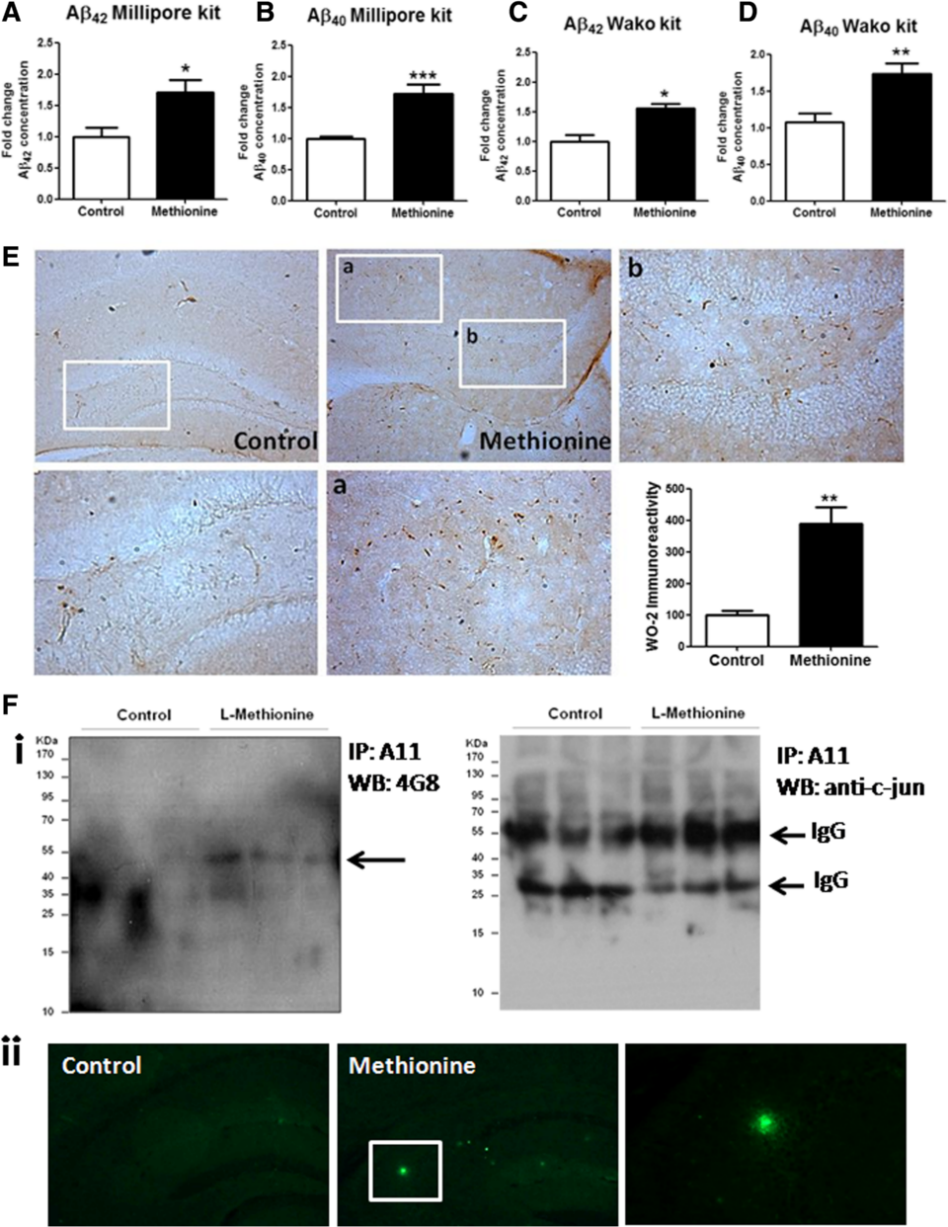
Accumulation of Aβ peptides and formation of Aβ oligomers are induced in animals treated chronically with L-methionine. Aβ_42_ and (**a** and **c**) Aβ_40_ (**b** and **d**) were examined by two different ELISA assay. **e** Histological slices were treated by immunohistochemistry with WO2 antibody to analyze Aβ levels and location. Hippocampus sections of control mice at 20x and 40x; L-methionine sections at 20x and 40x (**a** and **b**). The right panel shows the quantification (*n* ≥ 4) **f**) Immunoprecipitation of 100 μg of total proteins from the hippocampus with A11 antibody and western blot with 4G8 antibody and c-jun antibody (*i*). Histological slices were treated by immunofluorescence with 4G8 antibody to analyze Aβ levels and location (*ii*). Hippocampus sections of control mice at 20x and L-methionine sections at 20x and 40x). Bars are mean ± SEM. *P < 0.05, **P < 0.01, ***P < 0.001

Previous studies have shown that metabolites from the methionine-homocysteine cycle can enhance the activity of γ-secretase, a protease that cleaves APP [36]. Increased γ-secretase activity favors the amyloidogenic processing of APP, causing an increase of Aβ_40_ and Aβ_42_ in APP transgenic mice, as measured by the ELISA assay [34]. To test whether this effect occurred in our wild-type mice experimental groups, we measured the Aβ peptide accumulation. Hippocampal lysates from both groups (methionine-treated and non-treated) were used to detect both Aβ_42_ and Aβ_40_ levels by two different ELISA assays, obtained by Millipore [37] and a mouse-specific Aβ ELISA previously described [38]. The levels of both peptides increased significantly in methionine-treated mice relative to control mice (Fig. 2a, b, c and d). Additionally, using immunohistochemistry, we examined different sections of brain tissues from control and L-methionine mice using the WO2 antibody. Brain sections from L-methionine mice showed higher immunoreactivity than control mice did in the hippocampus, in agreement with the biochemical analysis (Fig. 2e), however we cannot discard that immunoreactivy observed may reflect also Aβ, APP, APP fragments, βCTF, or Aβ fragments. To determine the apparition of oligomeric Aβ species with the treatment, we performed immunoprecipitation assay of oligomers with the A11 antibody, which binds to a specific oligomeric conformation, and confirm that these oligomers were of Aβ using the 4G8 antibody in tris-Tricine gel. The western blot detects a 56 kDa band only in treated mice, suggesting the presence of amyloid oligomers in the hippocampus of methionine treated animals, which has previously been described as a toxic assembly of Aβ that contributes to cognitive decline [39]. As control, we did a western blot using the Aβ-unrelated antibody (anti-c-jun) to detect IgG and discard a possible reactivity of heavy and light chains in the experiment (Fig. 2f-i). Also, we performed an exploratory indirect immunofluorescence using the 4G8 antibody noticing the presence of fluorescent marks in the treated mice brain and not so in the control mice, suggesting the presence of non-fibrillar aggregates of Aβ peptides or senile plaques in methionine treated mice. (Fig. 2f-ii). According to these results, we decided to evaluate the presence of amyloid-β plaques in L-methionine and control mouse brains. Tissues were analyzed with Thioflavine-S staining in both the cortex and hippocampal sections CA1 and CA3 and in the dentate gyrus (DG). The staining did not show the presence of plaques in treated or control brain tissues (data not shown). Taken together, these results showed an increase in the levels of both Aβ peptides and Aβ oligomers, without presenting detectable plaques, as a direct or indirect consequence of chronic L-methionine overconsumption.

### Neuroinflammation

**Fig. 3:**
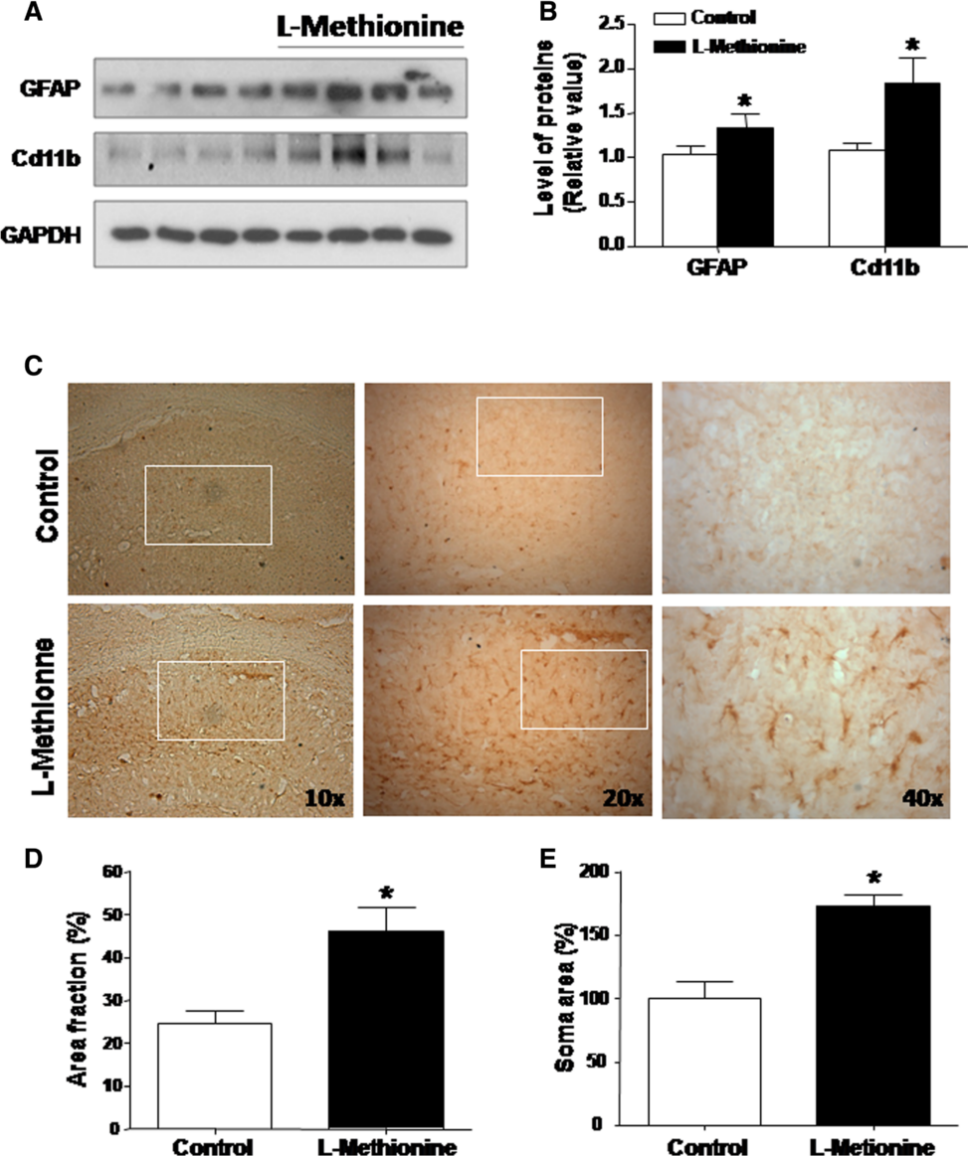
Increased astrogliosis in animals chronically treated with L-methionine. **a** Western blot measurements of GFAP and Cd11b in hippocampal lysates from L-methionine and control mice. **b** Densitometric analysis of **a**). **c** Histological slices were analyzed for glial fibrillar acid protein (GFAP) and observed under microscopy in the CA1 area of the hippocampus. Upper images show three different magnifications (10x, 20x and 40x) of control zones. Lower images show comparable zones of L-methionine-treated mice. **d** Quantification of the photographs. **e **The soma area of GFAP was calculated against total area of field (*n* ≥ 4) Bars are mean ± SEM. *P < 0.05, **P < 0.01, ***P < 0.001

Diverse studies have shown a link between methionine and the immuno-inflammatory activation, which is characteristic of neurodegenerative disease such as AD [40, 41]. To unveil whether a high level of L-methionine increases the brain inflammatory response, hippocampal slices were subjected to immunoblot and immune-cytochemical analysis. The astrogliosis marker GFAP [40, 41] and the microgliosis marker CD11b [40, 41] were evaluated in hippocampal lysates of the control and L-methionine groups (Fig. 3a). The levels of both markers were increased, as shown by densitometric analysis (Fig. 3b). Figure 3c shows representative photographs of the evaluated slices. The top row shows GFAP immunoreactivity in the hippocampi of control mice, and the bottom row shows the immunoreactivity in L-methionine-treated mice at different magnifications (10x, 20x and 40x). In every case, the white boxes indicate the area magnified from the photograph on the left. As shown in Fig. 3d, the fraction area of GFAP-positive hippocampal cells was significantly increased in L-methionine-treated mice. Additionally, the soma area of GFAP-positive cells was also significantly elevated (Fig. 3e). The immunohistochemical analyses were coincident with the data obtained from immunoblot analysis. These data show that an L-methionine-rich diet generates neuroinflammation and an increased activity of astrocytes and microglia in the hippocampus.

### Oxidative stress

**Fig. 4:**
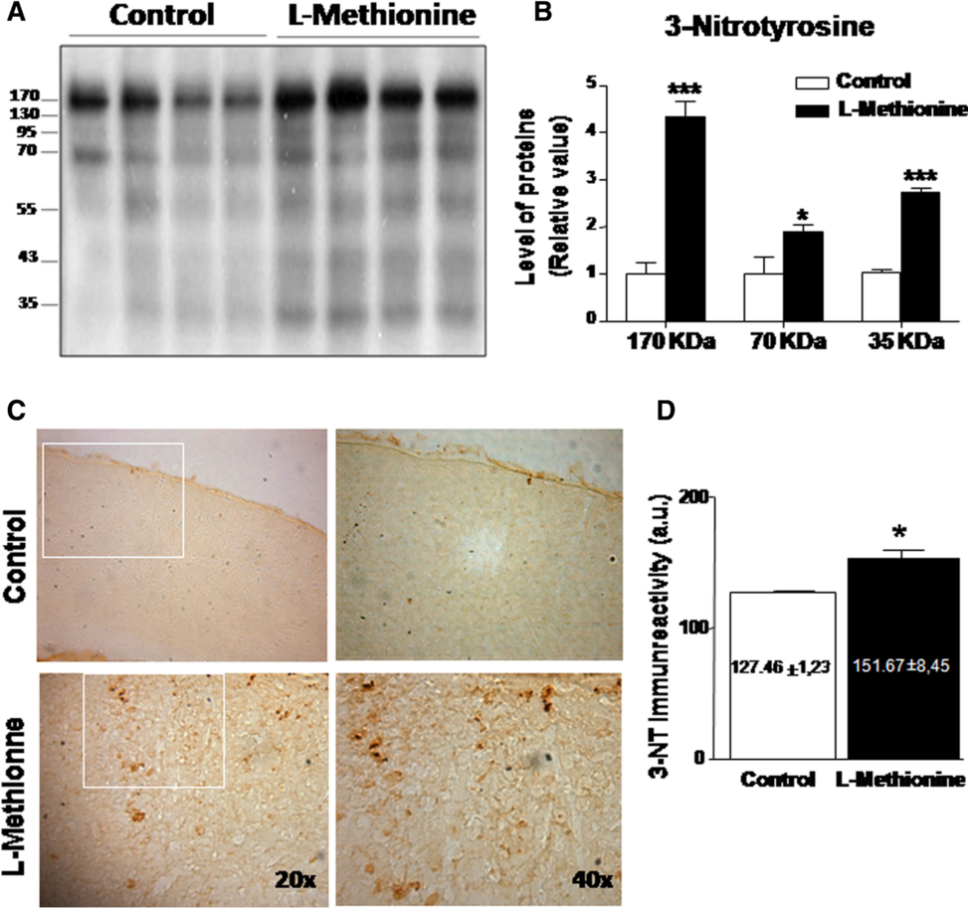
Proteins with 3-nitrotyrosylation are increased in L-methionine cortex and hippocampus samples. **a** Western blot of hippocampus lysates using 3-NT antibody. **b** Densitometric analysis of proteins at 170, 70 and 35 kDa, respectively. **c **Histological slices were treated to evaluate 3-nitrotyrosylation levels. The upper photographs show the control cortex, and the lower photographs show the L-methionine cortex at 20x and 40x. **d** Quantification of the photographs (*n* ≥ 4). Bars are mean ± SEM. *P < 0.05, **P < 0.01, ***P < 0.001

High levels of methionine and its metabolites could cause the deregulation of oxidative stress [42–44]. Therefore we evaluated the total levels of 3-nitrotyrosine (3-NT) in proteins. Immunoblot analyses of control and L-methionine mice showed that the levels of total proteins with 3-NT was increased in L-methionine-treated mouse hippocampi. Lanes 1–4 show control hippocampus lysates, and lanes 5-8 show L-methionine hippocampus extracts, with each lane extracted from different animals (Fig. 4a). Densitometric quantifications of the 170, 70 and 35 kDa proteins are shown in Fig. 4b. In every case, proteins of L-methionine-treated mice had more 3-NT than control animals did. Brain slices were subjected to immunohistochemical analysis using 3-NT antibody, confirming our previous findings. Figure 4c shows representative photographs at two magnifications (20x and 40x) of evaluated slices; the top shows the control, and the bottom shows the L-methionine mouse cortex. Quantification showed that 3-NT was significantly increased in the L-methionine mouse cortex compared with the control mouse cortex (Fig. 4d). These data support the idea that oxidative stress species are increased in the cortex and hippocampus of mice treated with an L-methionine rich diet.

### Synaptic protein loss

**Fig. 5:**
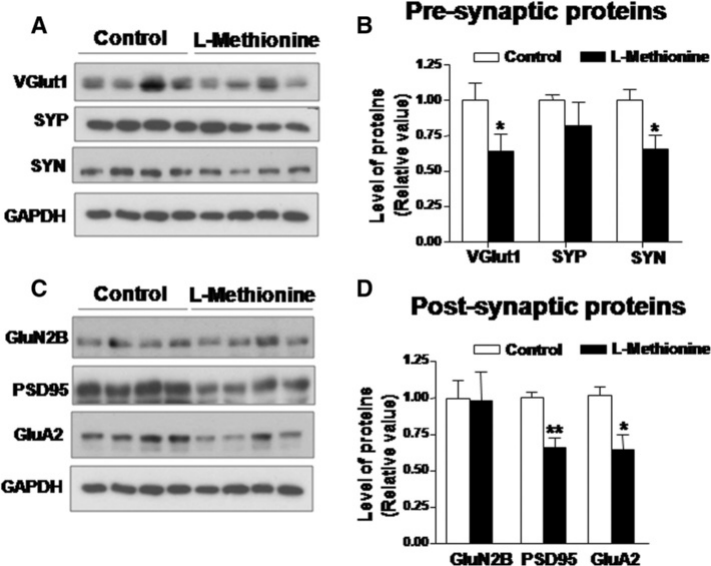
Chronic treatment with L-methionine decreases synaptic protein levels. **a** Different pre-synaptic protein levels were evaluated through western blot; lanes 1–4 are control samples and lanes 5–8 are methionine samples. **b** Quantification of **a**). **c** Western blot of different post-synaptic proteins with the same loading schema mentioned in **a**). **d** Densitometric analysis of **c**) (*n* ≥ 4). Bars are mean ± SEM. *P < 0.05, **P < 0.01, ***P < 0.001

Postmortem brains from patients with schizophrenia are characterized by a decrease in the dendritic spine density in the neurons of the prefrontal cortex [45]. Interestingly, the schizophrenic symptoms are exacerbated with L-methionine treatment [46] because the amino acid is apparently responsible for the decrease in dendritic spine density in the mouse frontal cortex [47]. In addition, in chronic schizophrenic cerebellar samples, a loss of synaptic but not cytoskeletal proteins was observed [48]. We have previously shown that in a double transgenic mice model of AD, a decrease in the levels of several synaptic proteins was part of the synaptic failure observed in the hippocampus and cortex of AD mice [49, 50]. Therefore, we were interested in evaluating the effect of chronic L-methionine treatment on these synaptic proteins. First we evaluated the following pre-synaptic proteins: vesicle glutamate transporter 1 (VGluT1), synaptophysin (SYP), and synapsin (SYN) (Fig. 5a). The levels of VGluT1 and SYN, but not SYP, were significantly decreased in animals treated with L-methionine (Fig. 5b). Then, we evaluated the post-synaptic proteins corresponding to AMPA receptor subunit 2 (GluA2), NMDA receptor subunit 2B (GluN2B) and the scaffold protein of the post-synaptic density, PSD-95 (Fig. 5c). Mice treated with high doses of L-methionine exhibited decreased levels of GluA2 and PSD-95 compared with control mice (Fig. 5d). These results indicate that chronic L-methionine treatment induces the loss of several pre-and postsynaptic proteins in the hippocampi of wild-type mice.

### Cognitive impairment

**Fig. 6:**
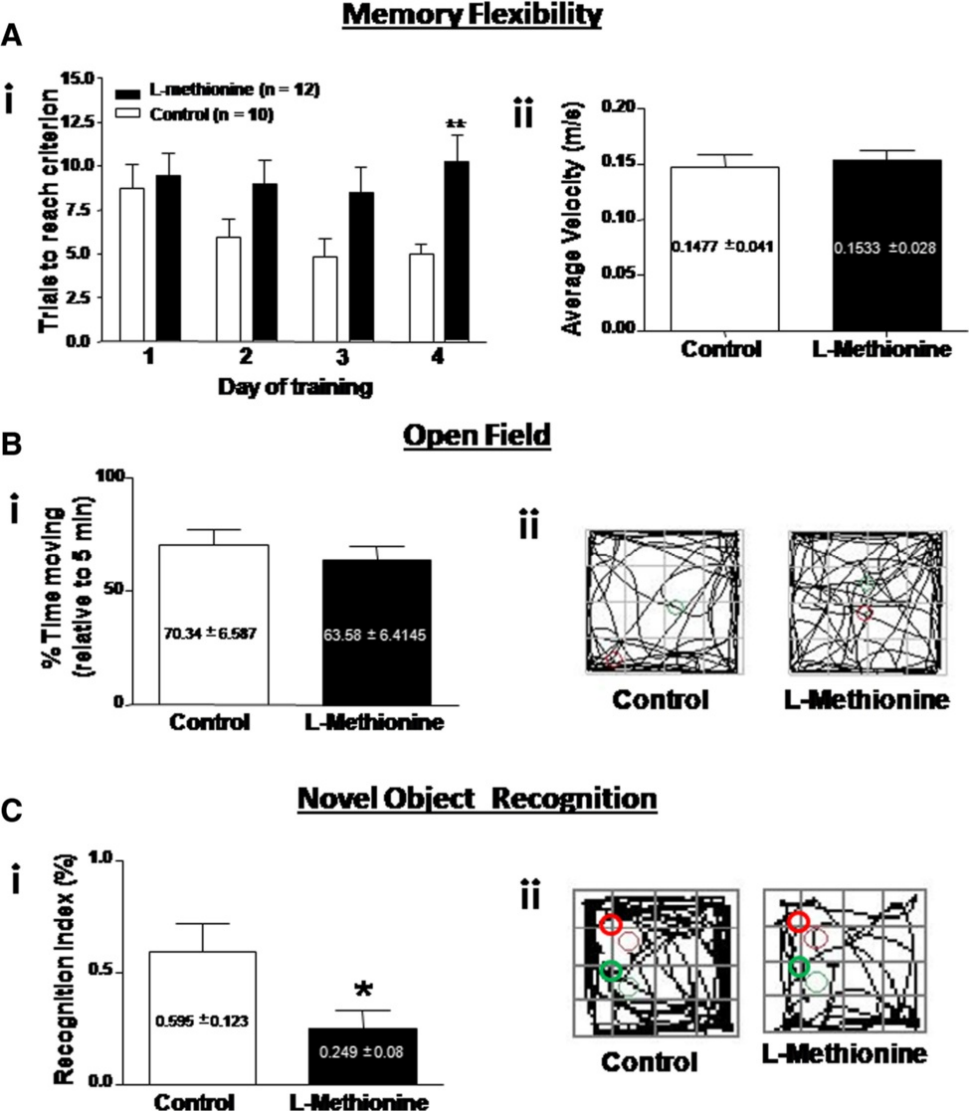
Memory is affected in mice treated with excess L-methionine for 10 weeks. **a** *i* Memory flexibility tests were performed for four consecutive days in the L-methionine group (black bars) and the control group (white bars). *ii *Measurements of average velocity. **b** *i* Open field tests were performed by the control and L-methionine groups. *ii* Representative trajectories of control and L-methionine mice in open field tests (*n* ≥ 12) **c** *i* The novel object recognition (NOR) test was performed by control (white bar) and L-methionine (black bar) animals. *ii* NOR representative traces of control and L-methionine mice; red circles indicate the position of the old object, while green circles indicate the position of the new object. Bars are mean ± SEM. *P < 0.05; **P < 0.01, ***P < 0.001

The progressive decline of cognitive abilities is a defining characteristic of AD [31]. It is not clear whether methionine and/or its metabolites is/are partly responsible for the cognitive impairment or are predictors of cognitive decline, as previously suggested [28, 51, 52]. Furthermore, several studies have shown that high intake of methionine could cause a loss of memory [28, 51]. To evaluate whether a methionine-enriched diet has an effect on behavior, particularly in learning and memory processes, we subjected control and L-methionine-treated animals to different tasks, including the memory flexibility (MF) test, a variant of the Morris water maze, and the Novel Object Recognition (NOR) test. Figure 6a (i) shows that the animals from the control group continuously decreased the number of trials required to complete the task and reach the criterion, whereas animals from the L-methionine group did not show the same behavior as the days passed. This difference was significant on the fourth day of training; Control mice needed an average of 5 trials to reach the criterion, when L-methionine animals required an average of 10, needing twice as control trials to reach the criterion of the test. This difference indicates that L-methionine mice had significant memory impairment. The average velocity in the maze is shown in Fig. 6a (ii). No difference between the groups was observed, so the treatments did not affect the motor activity of animals, and the data obtained exclusively reflect memory impairment. After two days of rest, animals were subjected to the NOR test. Figure 6c (i) shows that the animals from the L-methionine group spent ~35 % less time exploring the new object compared with control mice. The cognition index was measured as the percentage (%) of time that the mice spent exploring the new object versus the time that the mice spent exploring both objects. Then, to discard any anxious behavior that could have been developed during the diet change, we performed an open field (OF) test. Figure 6b (i) shows no change in the % of time between control and L-methionine mice. Representative OF tracks are shown in Fig. 6b (ii). Other parameters of the OF were quantified; L-methionine animals did not show any significant difference in the traveled distance, % of time in the center, % of time in the borders or the number of sections crossed compared with control animals (Additional file 4: Table S2). These data are important to validate any posterior behavioral assessments because anxiety could have altered the animals’ conduct. Overall, L-methionine-treated mice showed memory impairment in two different tests (MF and NOR), which indicated that a diet high in L-methionine triggered a decline in the cognitive capacities of wild-type mice.

### Dysfunction of *Wnt* signaling

**Fig. 7:**
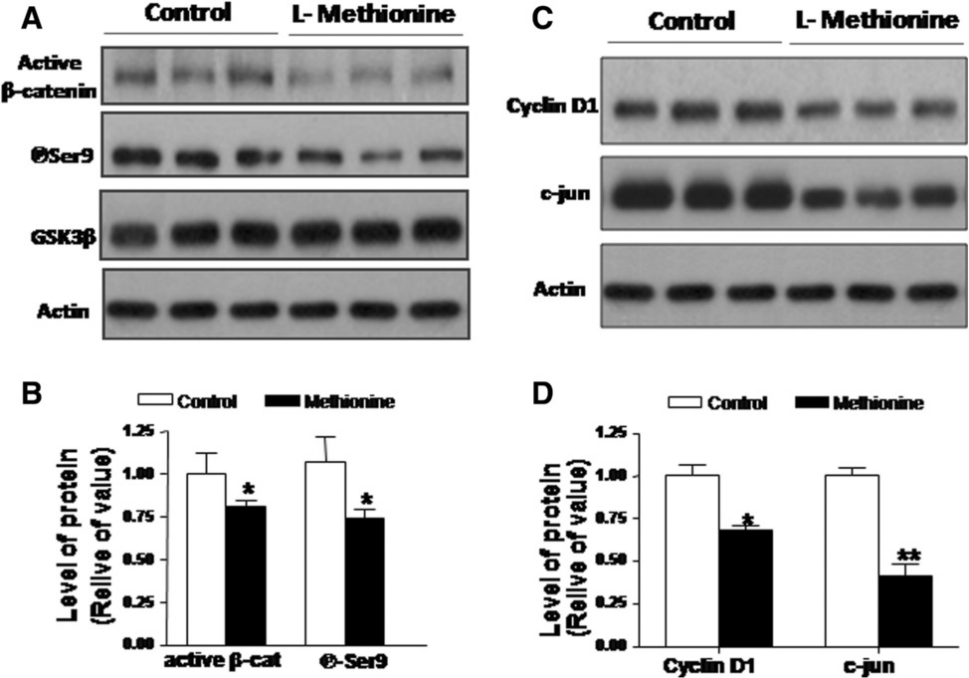
Methionine treatment induces a loss of the *Wnt* canonical pathway. **a** Representative western blots to show the levels of active β-catenin and the inhibited form of GSK-3β. **b** Densitometric analysis of **a**). **c** Western blot of the *Wnt* target genes Cyclin-D1 and c-jun. **d** Densitometric analysis of **c**) (*n* ≥ 4). Bars are mean ± SEM. *P < 0.05, **P < 0.01, ***P < 0.001

*Wnt* signaling plays an important role in the development of the nervous system and participates in the adult brain’s mediation and regulation of the structure and function of synapses [53, 54]. Over the years, several studies from our laboratory have strongly suggested a relationship between the loss of *Wnt* signaling and the neurotoxicity of Aβ, which is the main cause of the neuro-degeneration observed in AD [55–57]. Additionally, canonical *Wnt* signaling is necessary for object recognition memory [58]. Therefore, we evaluated whether an L-methionine-enriched diet affects canonical *Wnt* signaling. Interestingly, methionine causes a decrease in the active form of β-catenin levels (Fig. 7a) and an increase in the activity of GSK-3β, as indicated by the reduction in the levels of its inhibited enzymatic form (phospho-Ser-9) (Fig. 7a). Figure 7b shows the densitometric analysis of the above results. The activation of *Wnt* signaling leads to the transcriptional activation of *Wnt* target genes [59–61]. In contrast, in mice with an L-methionine-enriched diet, we observed a decrease in the levels of cyclin-D1 and c-jun. Interestingly, the decrease was almost 60 % compared with the control diet (Fig. 7c and d). These results suggest a loss of function of *Wnt* signaling in animals treated with L-methionine. Overall, this study shows that an L-methionine-enriched diet contributes to the appearance of Alzheimer’s-like neurodegeneration.

## Discussion

In the present work, we found that animals treated with an L-methionine-enriched diet exhibited an increase in the levels of Aβ_1-42_ peptide and its aggregation as well as increased *tau* phosphorylation, both hallmarks of AD. In addition, we found increased levels of neuroinflammation, oxidative stress and decreased synaptic proteins. Consistent with these effects, the L-methionine treated animals also showed clear cognitive deficits. Finally, for the first time, we described a loss in the components of the canonical Wnt signaling pathway. Together, these data demonstrate that increasing the levels of L-methionine intake might contribute to the development of Alzheimer’s-like neurodegeneration.

This is the first study to utilize chronic treatment with L-methionine for 12 weeks in wild-type mice and thereby focus on the neurobiological effects of L-methionine and its association with neurodegeneration, such as the one observed in AD. A few other studies regarding these effects have been published, although they were not as detailed as in this work and were not conducted in wild-type mice. Using a wide variety of experimental tools, we concluded that hyper-methioninemia has important neurotoxic effects on hippocampal components and function with characteristics of AD in wild-type mice.

In animals fed with an enriched methionine diet, we detected higher levels of *tau* phosphorylation and Aβ peptide load in hippocampal lysates. Because both features are considered hallmarks of AD, this finding led us to propose that L-methionine causes an AD-like disease. These data could explain the loss of synaptic proteins (Fig. 5) and the memory impairments (Fig. 6) described previously; however, we must be cautious because we cannot confirm that there is a causal relationship, only that there is a high correlation. According to the amyloid hypothesis, the production, aggregation and accumulation of Aβ peptides are the main features responsible for AD; [62], therefore, these processes could have triggered the synaptic failure, oxidative stress and neuroinflammation observed here. Interestingly, in the L-methionine-treated mice, only the epitopes T231 and S235 of the *tau* protein presented an increase in their phosphorylated levels (Fig. 1), whereas PHF1 and AT8 were unaffected. These epitopes, T231 and S235, have been associated with the triggering process of *tau* aggregation into neurofibrillary tangles [35]. In contrast, AT8 and PHF1 are associated with the process of aggregation and accumulation of paired-helical filaments that form the neurofibrillary tangles [63]. These results suggest that L-methionine would deregulate *tau* kinases or phosphatases, thereby favoring the dissociation of *tau* from the microtubules, which is an initial process in the early stage of AD.

As mentioned previously, one of the key aspects of AD corresponds to Aβ peptide accumulation and the formation of Aβ oligomeric structures [64]. In our results, we observed an increase in both the Aβ species levels and Aβ oligomers (Aβo) presence in the hippocampi of animals treated with a diet high in L-methionine compared with control mice (Fig. 2). One intriguing aspect was the fact that our results show an increase in the Aβ_40_ and Aβ_42_ species levels on the hippocampus of L-methionine mice by using two different kits and also demonstrate the presence of 54-KDa Aβ species, which have been described as synaptotoxic Aβo structures (Fig. 2e), and this could have been the main triggering factor for the memory loss observed in these treated animals (Fig. 1). However, we were not able to visualize Aβ plaques in the Th-S staining of brain slices from any group of animals (data not shown). We attributed this result to the fact that wild-type rodents are not able to form senile plaques unless they are induced by an exogenous nucleating factor, such as acetylcholinesterase [65]. Previous studies showed that in an APP mice model of AD, consumption of a methionine-rich diet for 10 weeks increased the levels of total brain Aβ peptide and phosphorylated *tau* in the brain [34]. However, those authors did not study a specific brain region, and our results were obtained specifically in the hippocampus; moreover, we treated the animals for 12 weeks instead of 10. These variables and the differences in the time periods probably explained the minor differences between our results.

It is known that the augmented nitrotyrosine content in proteins is related to neurodegenerative diseases such as Parkinson’s, amyotrophic lateral sclerosis, and AD [66]. S-nitrosylation (SNO) is a post-translational modification caused by nitric oxide radicals reacting with cysteine thiols [42]. Here, we examined the total levels of protein nitrotyrosylation. In previous studies, Foster et al. [67] described a considerable number of proteins that suffered increased nitrosylation in AD [67, 68]. In fact, drugs that decrease SNO, such as deprenyl and rosiglitazone, have been reported to ameliorate Type2 diabetes and neurodegeneration. Interestingly, our group previously reported the beneficial effects of rosiglitazone in a transgenic mouse model of AD [50]. Although we did not investigate the cause of the SNO increase in the present study, a possible explanation for this increase might be related to the over-activation of the NMDA receptor: it has been found that the over-activation of this receptor causes deregulation in SNO, which is an interesting fact in the context of neurodegenerative diseases. This increase in SNO correlates with the increased astrocyte (GFAP) and microglia activation (Cd11b), which indicates neuro-inflammatory response; this relationship has been described separately by different groups [69].

Moreover, when different synaptic proteins were evaluated, we found that VGluT1, SYN, PSD-95 and GluA2 proteins were diminished in hippocampal extracts from animals with chronic treatment with L-methionine (Fig. 4). These results are consistent with studies that have demonstrated a decrease in synaptic proteins in brain samples from schizophrenic patients [48] and the loss of dendritic spines caused by L-methionine treatment [47]. Similar decreases have been reported in the hippocampus and cortex of double transgenic mice models of AD [49, 50, 70].

Synaptic plasticity, specifically at the hippocampus, has been strongly related to memory acquisition [71]. To evaluate memory, we conducted two different tests that evaluate hippocampus-dependent memory: the Morris water maze to test spatial memory flexibility and the Novel object recognition test to evaluate contextual memory. The memory flexibility tests showed that the L-methionine group was constant in its performance over all days, whereas the control group constantly decreased the number of trials required to reach the criterion. Because the average velocity was not different between groups, we can discard any motor activity failure caused by the treatment. Impairments in both spatial and contextual memory were observed in the L-methionine-treated group, as shown by the significant reduction in the novel recognition index. Anxiety and motor activity impairment were completely discarded by the results of the open field test, in which there was no significant difference between the control and L-methionine groups (Fig. 6). The increase in the production and aggregation of Aβ, together with the increased level of oxidative stress, inflammation, *tau* phosphorylation and loss of *Wnt* signaling, can explain the memory failure observed in the L-methionine group.

With respect to methionine, is important to recall that its metabolic product is homocysteine, the levels of which also increase with a high methionine diet [72]. Thus, homocysteine might have had a significant role in this alteration. Homocysteine is an aminoacid that is not present in the diet, and it can only be synthesized from methionine or another intermediate of the methionine cycle. This cycle begins with the addition of adenosine to the sulfur group of methionine by methionine adenosyltransferase (MAT), which activates the adjacent methyl group to form S-Adenosyl Methionine (SAM). Then, the process continues with the removal of the previously activated methyl group of SAM to produce S-Adenosyl Homocysteine (SAH), which is performed by the Zn-dependent methyl transferase, an enzyme that is responsible for important methylations of DNA and other molecules. Subsequently, the S-Adenosyl Homocysteine Hydrolase (SAHH) removes the adenosine molecule of SAH, which is converted into Homocysteine (Hcy) [73]. When this metabolism is disrupted and in the case of an overload of L-Methionine, many alterations could occur, such as Hiperhomocysteinemia, which is a condition that is characterized by high levels of homocysteine in the blood and is present in several disease conditions [74]. Moreover, several studies have found a correlation between hyperhomocysteinemia and AD [75, 76]. Unfortunately, the mechanism by which homocysteine is involved in AD is still unclear. There is some evidence that it could be involved in oxidative stress [77], endoplasmic reticulum stress [78] neuronal DNA damage [79], the enhancement of β-amyloid peptide-mediated vascular smooth muscle toxicity, demethylation [80] or Aβ elevation [81].

Nonetheless, it is not only homocysteine that can alter neural function: another metabolite of methionine cycle that can also participate is S-adenosylhomocysteine (SAH). SAH binds to methyltransferase enzymes, which causes their inhibition [82]. This inhibition could involve the Aβ peptide because the gene of the amyloid precursor protein (APP) is highly methylated, and it has been observed that a decrease in these methylations promotes the extracellular deposition of Aβ peptides [83]. In fact, new studies show that the inhibition of protein phosphatase 2A methyltransferase (PPMT) caused by hyperhomocysteinemia promotes *tau* and leads to APP deregulation [84]. Moreover, it is also known that homocysteic acid, an agonist for N-methyl-D-aspartate (NMDA) but a partial antagonist of the glycine coagonist site, can be responsible for some alterations, such as excitotoxicity and apoptosis [85]. Given these findings, we cannot affirm that all the effects observed with a high methionine level are caused by an increase in the levels of homocysteine or its related metabolites.

A strong relationship between the loss of function of *Wnt* signaling and the neuronal dysfunction observed during AD progression has been established [56, 70, 86–88]. Several studies have demonstrated that *Wnt* signaling components are altered in the AD brain: (1) β-catenin levels are reduced in patients carrying presenilin-1-inherited mutations [89]; (2) Exposing cultured hippocampal neurons to exogenous Aβ results in the inhibition of canonical *Wnt* signaling [90]; (3) Dickkoff-1 (*Dkk1*), a *Wnt* antagonist, is induced by Aβ in hippocampal neurons [87] and is able to reduce the amount of synaptic proteins [91]; (4) apo-lipoprotein E (apoEε4), a major genetic risk factor for AD, inhibits canonical *Wnt* signaling [92]; (5) a common genetic variation within the low-density lipoprotein receptor-related protein 6 (LRP6) leads to AD progression [93]; a decrease in LRP6 levels is observed in AD patients [94]; and (6) there are certain polymorphisms in the clusterine gene, according to GWAS, that may modulate the risk for late-onset AD [95], and induces *Dkk-1* expression [88]. Therefore, considering the above roles of *Wnt* signaling, it is highly possible that a decrease or deregulation of its components may contribute to the synaptic dysfunction that is characteristic of early stages of AD [70, 96]. In addition, canonical *Wnt* signaling regulates hippocampal development, synaptogenesis and synaptic plasticity [96, 97]. Canonical ligands are released, and their levels are regulated by synaptic activity in the hippocampus, which suggests an essential role for canonical *Wnt* signaling in the process of learning and memory [98-100]. Indeed, other studies have demonstrated that canonical *Wnt* signaling is necessary for hippocampal object recognition memory consolidation [58]. In the present work, a high-methionine diet caused a decrease in β-catenin and the inhibition of GSK-3β activity. Moreover, a reduction in the protein levels of the *Wnt* target genes cyclin-D1 and c-jun was observed, which indicated a decrease in the activity of the entire *Wnt* signaling pathway (Fig. 7). These results suggest that L-methionine induces a loss of *Wnt* signaling function, a situation reminiscent of what has been observed in AD [70, 88, 101].

## Conclusions

In the present work, we studied the effect of chronic treatment with a methionine-enriched diet on AD hallmarks, including Aβ accumulation, *tau* phosphorylation and memory impairment. We concluded that methionine affects brain function and induces memory impairment in mice, thereby generating Alzheimer’s-like disease in wild-type animals.

## Methods

### Antibodies

Primary antibodies used: mouse monoclonal anti-PSD95 clone K28/43, obtained from Antibodies Inc (UC Davis/NIH NeuroMab Facility), mouse anti-GluN2B (clone N59/36; UC Davis/NIH NeuroMab Facility), mouse anti-GluA2 (clone L21/32; UC Davis/NIH NeuroMab Facility), goat anti-synaptophysin (sc-7568 Santa Cruz Biotechnology, Inc.), mouse anti-Aβ 4G8 (Covance, Princeton, USA), mouse anti-Aβ WO2 (MABN10, Anti-Amyloid β Antibody, clone W0-2), conformational anti-amyloid oligomer A11 (AB9234, Millipore), Anti-β-Actin clone AC-15 (A1978, Sigma Aldrich), rabbit anti-GFAP (M0761, Dako, Cytomation) and anti-3-nitrotyrosine (A21285, Invitrogen).

### Animals and treatments

A total of 12 wild-type (C57/BL6) female animals were treated with 8.2 g/kg L-methionine placed in their drinking water for 12 weeks. L-methionine concentration was determined based on the total water intake of mice to achieve high levels of the amino acid in the plasma without reaching toxic levels, which could lead to organ failure [30]. In fact, once a week throughout the course of treatment, we monitored the animals’ body weight and water intake. These results are shown in Additional file 1: Figure S1. To further analyze the health of the mice, lipid and hepatic parameters were measured at the end of the treatment period. No significant differences were observed between the control and L-methionine-treated groups (Additional file 4: Table S1). In parallel, a second group of control animals (C57/BL6) were treated for the same time period with regular tap water (*n* = 12). Water supplies with or without methionine were changed three times a week and consumption was measured.

### Immunoblotting

The hippocampi from treated or control mice were dissected on ice and immediately frozen at–150 °C or processed as detailed previously [49, 102]. Briefly, slices were homogenized in RIPA buffer (50 mMTris-Cl, pH 7.5, 150 mMNaCl, 1 % NP-40, 0.5 % sodium deoxycholate, and 1 % SDS) supplemented with a protease inhibitor cocktail (Sigma-Aldrich P8340) and phosphatase inhibitors (50 mMNaF, 1 mM Na_3_VO_4_ and 30 μM Na_4_P_2_0_7_) using a Potter homogenizer and were then passed sequentially through different caliber syringes. Protein samples were centrifuged at 14,000 rpm at 4 °C twice for 15 min. The protein concentration was determined using the BCA Protein Assay Kit (Pierce Biotechnology, Rockford, IL). Twenty- and forty-microgram samples were resolved by 10 % SDS-PAGE and transferred to a PVDF membrane. The reactions were followed by incubation with anti-mouse, anti-goat or anti-rabbit IgG peroxidase-conjugated antibody (Pierce, Rockford, IL) and developed using an ECL kit (Western Lightning Plus ECL, PerkinElmer).

### Determination of Aβ peptides

To determine the concentrations of Aβ peptides, two Sandwich Enzyme-linked Immunosorbent Assay (ELISA) specific for Aβ_1-40_/Aβ_1-42_ were used as previously described. (EZBRAIN40, EZBRAIN42; EMD Millipore Corporation, Billerica, MA and ELISA kit (Catalog Number: 294-62501) Wako Human/Rat(Mouse) β-Amyloid (40) ELISA Kit (Catalog Number: 294-62501), and Wako Human/Rat(Mouse) β-Amyloid (42) [37, 38]. Briefly, hippocampal homogenates of control or methionine-treated animals were diluted to 2 μg/μl in homogenization buffer containing protease inhibitors. Approximately 50 μl of crude homogenate was prepared to measure Aβ_1-40_/Aβ_1-42_ levels according to the manufacturer’s instructions. Plates were read at the respective light wave on a Metertech 960 ELISA Analyzer. To detect soluble Aβ peptides for western blot, samples from the hippocampus were centrifuged at 20,0000 *g* for 1 h and the protein concentration from each supernatant was determined using the BCA Protein Assay Kit (Pierce Biotechnology, Rockford, IL).

### Immunoprecipitation assay

Protein extracts were obtained from hippocampal tissue lysed in RIPA buffer supplemented with protease and phosphatase inhibitors. Aβ immunoprecipitation was performed using rabbit anti-A11 antibody using the protein G-Agarose. Immunocomplexes were analyzed by Tris-tricine gels, transferred to PVDF membranes and immunoblotted with a mouse anti-4G8 antibody.

### Immunohistochemistry

The procedure was performed as previously described [102]. Animals were fixed with 4 % paraformaldehyde, and their brains were dissected and maintained in 4 % paraformaldehyde for 2 weeks to ensure proper fixation before being placed in a PBS-20 % sucrose solution for 48 h. Brain slices (30 μm) were obtained using a Leica CM1850 Cryostat and maintained in Olmos solution at 4 °C. Free-floating cytochemistry procedures were performed as follows: slices of tissue were treated with H_2_O_2_ in PBS to eliminate endogenous peroxidase activity, then washed with PBS and blocked with PBS/BSA (2 mg/ml) at room temperature for 60 min to avoid non-specific binding. Slices were incubated with glial fibrillar acidic protein, 3-nitrotyrosine, AT8 or WO2 antibodies in PBS + 0.2% Triton X-100, for 12 h at 4 °C. After washing with PBS + 0.2% Triton X-100, the sliceswere incubated with secondary antibodies coupled to HRP for 60 min at room temperature. Slices were incubated with 0.06 % diaminobenzidine in PBS for 15 min, and 0.01 % H_2_O_2_ was added. For all assays, *n* ≥ 4 animals were used, and three slices for each mouse were analyzed. Stained brain sections were photographed using an Olympus BX51 microscope (Tokyo, Japan) coupled to a Micro-publisher 3.3 RTV camera (QImaging). The luminance of the incident light and the time of exposure were calibrated to assign pixel values ranging from 0 to 255 in the RGB image (no-light to full-light transmission), which were used along all preparations. The photographs were captured with 20x and 40x objectives. The images were analyzed using theImage-J software (NIH) to measure the total area occupied by the cortex or hippocampus.

### Immunoflourescence

Immunofluorescence in brain slices was performed as described previously [103, 104]. Slices were washed three times in ice-cold PBS and then permeabilized for 30 min with 0.2 % Triton X-100 in PBS. After several rinses in ice-cold PBS, the slices were incubated in blocking solution (3 % bovine serum albumin in PBS) for 1 h at room temperature followed by an overnight incubation at 4 °C with mouse anti-4G8 antibody (Covance, Princeton, USA). After primary antibody incubation, the slices were extensively washed with PBS and then incubated with GFP-conjugated secondary antibodies (Molecular Probes, Carlsbad, USA) for 2 h at 37 °C. Then, slices were mounted with mounting medium on gelatin-coated slides and analyzed by fluorescence microscopy. The images were analyzed using NIH Image J software.

### Behavioral tests

*Memory flexibility* (MF) tests were performed as previously described [50]. Briefly, a circular white pool was prepared with non-toxic white paint plus a hidden platform (diameter: 9 cm) in four quadrants; the animals were pre-trained in this pool for 60 s (s), one day before the actual testing began. The water temperature was kept between 18 and 20 °C. To acclimate the animals to the room and the swimming strategy, the pool was used without the platform. Then, animals were subjected to testing for 4 consecutive days with a maximum of 15 trials per day. Every day, the platform position in a quadrant was changed. Testing stopped when the animal reached the platform on three consecutive trials with an average of 20 s (s) or less. Data are presented as the number of trials after which animals reached the criteria.

*Open field* (OF) tests were performed two days after the memory flexibility tests. The animals were individually placed at the center of a 72 x 72 x 32 cm white acrylic boxand left to freely move within it for 10 min. For all behavioral tests, data were gathered and analyzed with a video tracking system (HVS Imagen, UK). Room temperature was maintained at 20 °C.

*Novel object recognition* (NOR) tests were performed a day after the OF test in a 38 x 38 x 32 cm white acrylic box [105]. Animals were pre-trained to habituate to the box for two consecutive days, without objects present. For testing, animals were placed individually at the center of the box in the presence of two identical objects (old objects) for 10 min. After that period, the box and objects were cleaned with 50 % methanol solution. The animal was later (after 2 h) exposed to one of the old objects and a new object of a different shape and color than the old object, and the box and objects were cleaned again to continue with the next animal. The recognition index was calculated as the time spent exploring the new object divided by the time exploring both objects.

### Biochemical profile (lipid and hepatic enzymes)

Anticoagulant-treated serum samples were obtained from the blood of treated and control mice. Plasma was separated through centrifugation and sent to Barnafi-Krause Laboratories (Santiago, Chile) and analyzed.

### Statistical analysis

Data analysis was carried out using the Prism software (GraphPad Software Inc.). The results were expressed as the means ± standard error. For statistical analysis, normally distributed data were analyzed by one-way ANOVA with post hoc tests performed using Tukey’s test. Non-normally distributed data were analyzed by the Kruskal-Wallis test and post hoc tests were performed using Dunn’s test. Behavioral data were analyzed using the non-parametric t-test.

## Additional files

Additional file 1: Figure S1. Water consumption and body weight are not affected by a high-L-methionine diet. **A**) Water intake was measured every 3 days during all 12 weeks of treatment, and the average water consumption was calculated per week with 3 measurements each week. L-methionine mice had lower levels of water intake than did control mice, but these levels stayed in the normal range (2.5 ml per animal/day) without being dehydrated in all 12 weeks of treatment. **B**) Body weight was measured one day a week on the same day and hour every time. Although L-methionine mice had a lower average body weight than did control mice, body weight gain was normal in both **p* < 0,05. (TIFF 81 kb) Additional file 2: Table S1. Levels of lipids and hepatic enzime profile in mice treated with L-methionine rich diet. (TIFF 85 kb) Additional file 3: Figure S2.
*tau* phosphorylation at the AT8 epitope was not significantly altered in L-methionine mice. **A**) Histologically fixed 30-μm slices from control and L-methionine mice brains were analyzed by immunocytochemistry to analyze the presence of AT8-positive cells that detect *tau* phosphorylation at Thr205. (Left) Cortex and hippocampus sections of control mice;(right) L-methionine brain sections, both at 20x. **B**) Quantification of the photographs did not show any significant difference on this epitope of *tau* phosphorylation between treated and control mouse tissues. (TIFF 533 kb) Additional file 4: Table S2. Open field test in mice with a high L-methionine diet. (TIFF 72 kb)

## Abbreviations

Aβ: Amyloid-β
SAM: S-adenosyl-methionine
MS: Methionine synthase
AD: Alzheimer’s Disease
NOR: Novel Object Recognition
OF: Open Field
MF: Memory Flexibility
%: Percentage
DG: Dendrate gyrus
VGluT 1: Vesicular glutamate transporter 1
SYP: Synaptophysin
SYN: Synapsin
3NT: 3-nitrotyrosine
SNO: S-nitrosylation
Aβo: Aβ oligomers
Hcy: Homocysteine
NMDA: N-methyl-D-aspartate
