# Supplementary figures and images for: Is L-methionine a trigger factor for Alzheimer’s-like neurodegeneration?: Changes in Aβ oligomers, tau phosphorylation, synaptic proteins, Wnt signaling and behavioral impairment in wild-type mice
Source: Mol Neurodegener. 2015 Nov 21;10:62. doi: 10.1186/s13024-015-0057-0 (PMC4654847; doi:10.1186/s13024-015-0057-0)

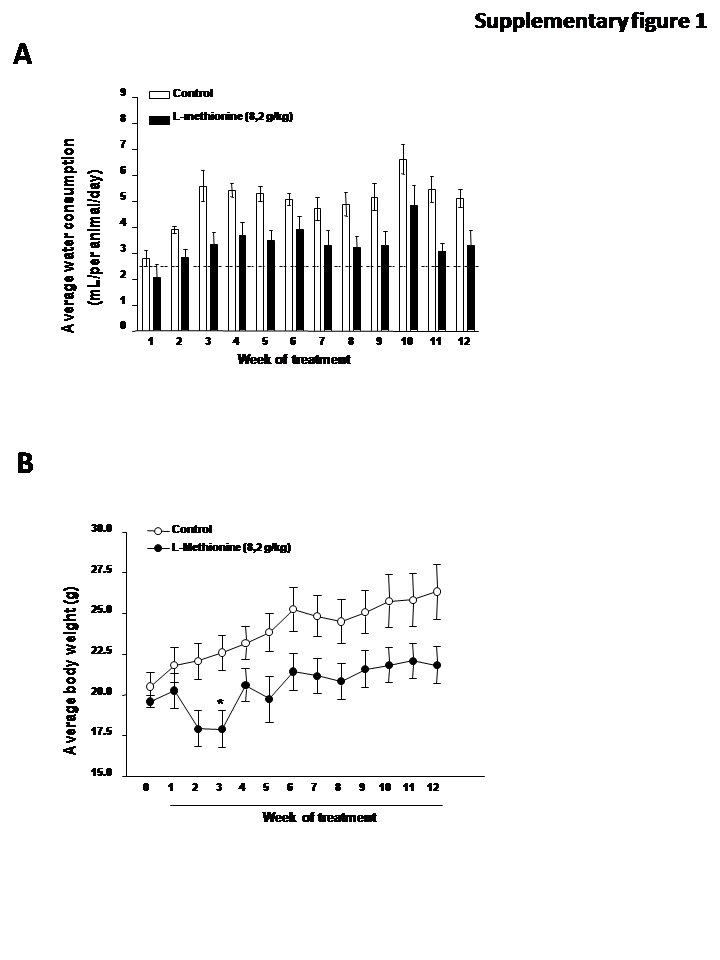

Supplement: Additional file 1: Figure S1. — Water consumption and body weight are not affected by a high-L-methionine diet. A) Water intake was measured every 3 days during all 12 weeks of treatment, and the average water consumption was calculated per week with 3 measurements each week. L-methionine mice had lower levels of water intake than did control mice, but these levels stayed in the normal range (2.5 ml per animal/day) without being dehydrated in all 12 weeks of treatment. B) Body weight was measured one day a week on the same day and hour every time. Although L-methionine mice had a lower average body weight than did control mice, body weight gain was normal in both *p < 0,05. (TIFF 81 kb) [file 13024_2015_57_MOESM1_ESM.tiff]

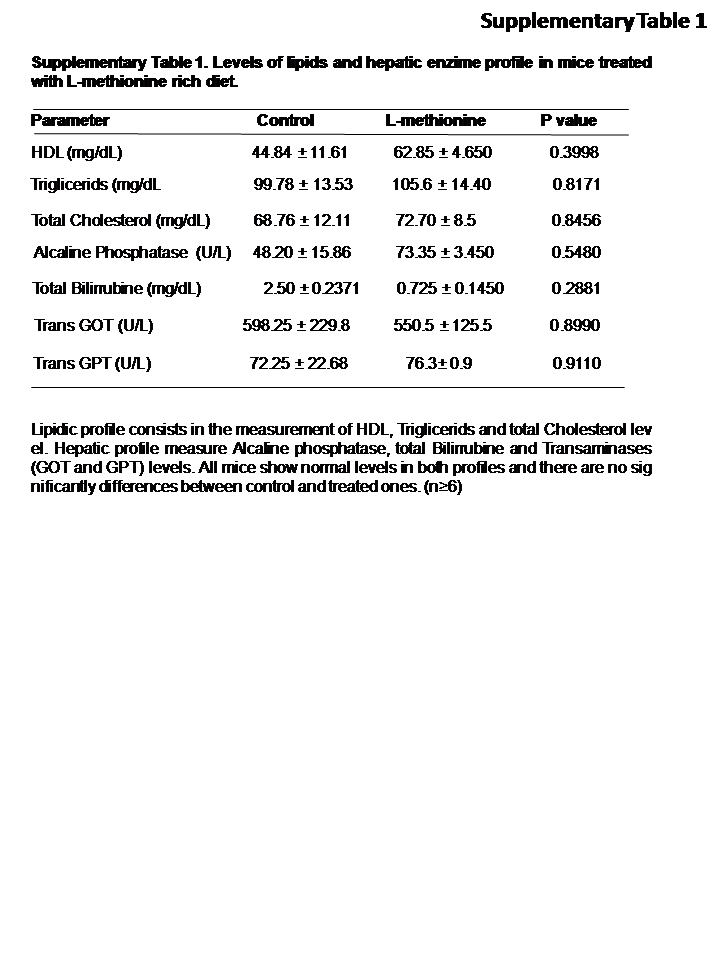

Supplement: Additional file 2: Table S1. — Levels of lipids and hepatic enzime profile in mice treated with L-methionine rich diet. (TIFF 85 kb) [file 13024_2015_57_MOESM2_ESM.tiff]

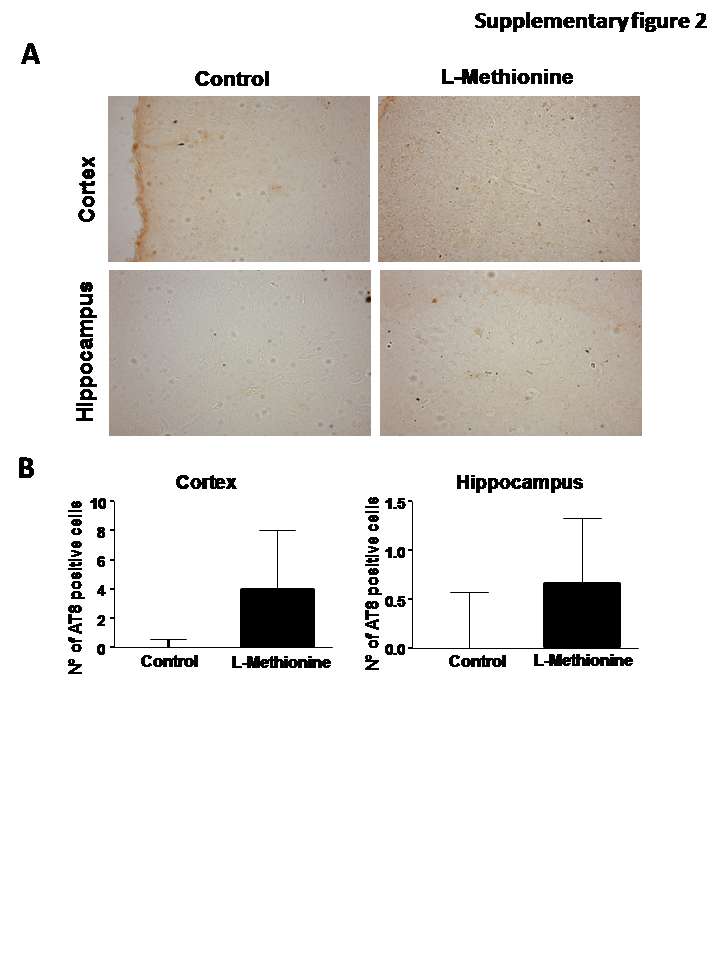

Supplement: Additional file 3: Figure S2. — tau phosphorylation at the AT8 epitope was not significantly altered in L-methionine mice. A) Histologically fixed 30-μm slices from control and L-methionine mice brains were analyzed by immunocytochemistry to analyze the presence of AT8-positive cells that detect tau phosphorylation at Thr205. (Left) Cortex and hippocampus sections of control mice;(right) L-methionine brain sections, both at 20x. B) Quantification of the photographs did not show any significant difference on this epitope of tau phosphorylation between treated and control mouse tissues. (TIFF 533 kb) [file 13024_2015_57_MOESM3_ESM.tiff]

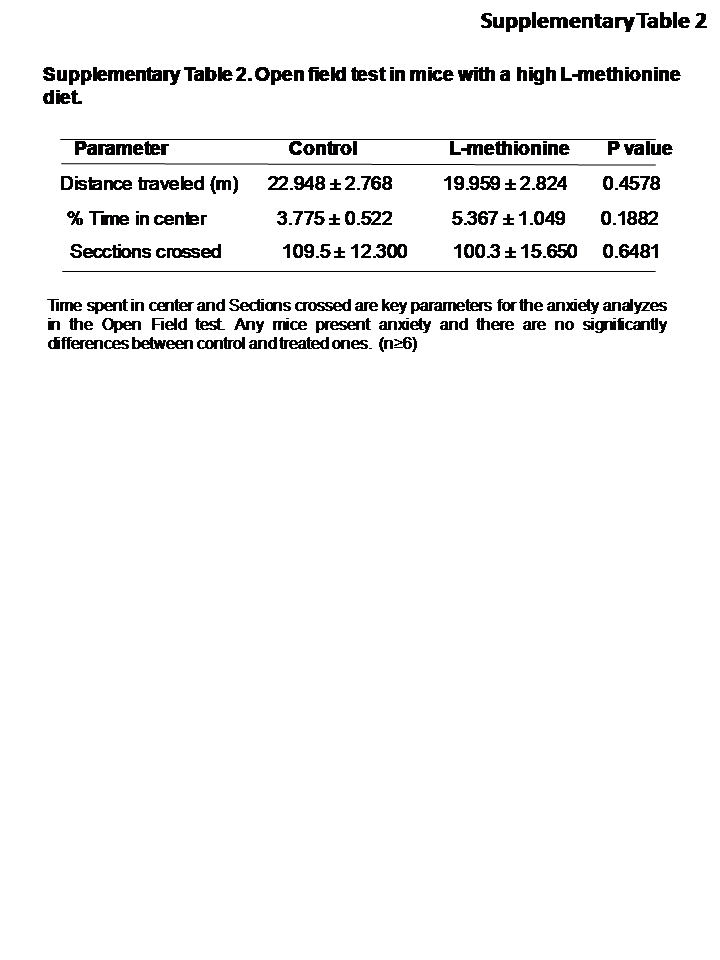

Supplement: Additional file 4: Table S2. — Open field test in mice with a high L-methionine diet. (TIFF 72 kb) [file 13024_2015_57_MOESM4_ESM.tiff]
